# Burst Suppression During General Anesthesia and Postoperative Outcomes: Mini Review

**DOI:** 10.3389/fnsys.2021.767489

**Published:** 2022-01-07

**Authors:** Niti Pawar, Odmara L. Barreto Chang

**Affiliations:** Department of Anesthesia and Perioperative Care, University of California, San Francisco, San Francisco, CA, United States

**Keywords:** burst suppression, electroencephalogram–EEG, geriatric, postoperative delirium (POD), cognitive decline, postoperative outcomes

## Abstract

In the last decade, burst suppression has been increasingly studied by many to examine whether it is a mechanism leading to postoperative cognitive impairment. Despite a lack of consensus across trials, the current state of research suggests that electroencephalogram (EEG) burst suppression, duration and EEG emergence trajectory may predict postoperative delirium (POD). A mini literature review regarding evidence about burst suppression impact and susceptibilities was conducted, resulting in conflicting studies. Primarily, studies have used different algorithm values to replace visual burst suppression examination, although many studies have since emerged showing that algorithms underestimate burst suppression duration. As these methods may not be interchangeable with visual analysis of raw data, it is a potential factor for the current heterogeneity between data. Even though additional research trials incorporating the use of raw EEG data are necessary, the data currently show that monitoring with commercial intraoperative EEG machines that use EEG indices to estimate burst suppression may help physicians identify burst suppression and guide anesthetic titration during surgery. These modifications in anesthetics could lead to preventing unfavorable outcomes. Furthermore, some studies suggest that brain age, baseline impairment, and certain medications are risk factors for burst suppression and postoperative delirium. These patient characteristics, in conjunction with intraoperative EEG monitoring, could be used for individualized patient care. Future studies on the feasibility of raw EEG monitoring, new technologies for anesthetic monitoring and titration, and patient-associated risk factors are crucial to our continued understanding of burst suppression and postoperative delirium.

## Introduction

**FIGURE 1:**
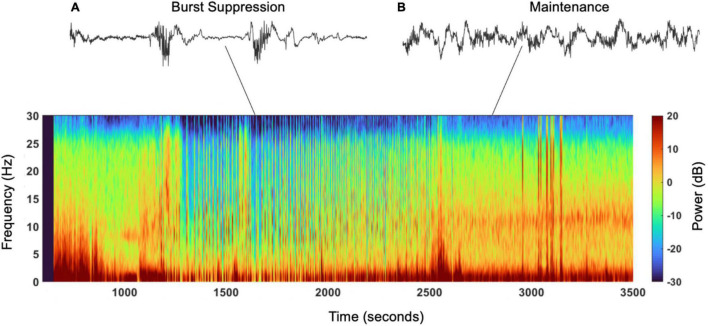
Spectrogram and EEG raw traces. Suppression episodes during burst suppression in the spectrogram show as periods of blue, vertical lines. **(A)** Electroencephalogram trace showing burst suppression, burst of activity followed by interburst periods of suppression (seen in the spectrogram from 1,250 to 2,000s). **(B)** Electroencephalogram trace showing oscillation/activity maintained under under general anesthesia (seen in the spectrogram from 2,500 to 3,500).

Burst suppression was first discovered by Derbyshire et al. (1936) and consists of alternating episodes of isoelectric flat EEG periods with bursts of slow waves, including systemic and quasiperiodic variation where high voltage and isoelectric periods have variations between and within bursts (Figure 1) (Swank and Watson, 1949; Niedermeyer et al., 1999; Ching et al., 2012; Amzica, 2015; Purdon et al., 2015b). Burst suppression has been identified in hypothermia, coma, early infantile epileptic encephalopathy, and in general anesthesia—the focus of this review (Shanker et al., 2021). There are many controversies about burst suppression, including its relation to postoperative delirium (POD)—the postoperative onset of acute change from baseline attention, fluctuating awareness, and disturbances in cognition representative of acute brain failure (American Geriatrics Society Expert Panel on Postoperative Delirium in Older Adults, 2015). The American Geriatrics Society recognizes POD as the most common surgical complication in older adults, occurring in up to 50% of patients after surgery. POD results in longer hospital stays, increased need for long-term care, loss of functional independence, reduced cognition, and death (American Geriatrics Society Expert Panel on Postoperative Delirium in Older Adults, 2015). It costs about $150 billion in the U.S. annually, even though it is preventable in up to 40% of patients (American Geriatrics Society Expert Panel on Postoperative Delirium in Older Adults, 2015). Furthermore, POD results in increased healthcare cost, thus its prevention has been declared a public health priority; in July 2010, the National Institute for Health and Clinical Excellence released a guideline addressing diagnosis, prevention, and management of delirium (National Clinical Guideline Centre (Uk), 2010). However, the link between burst suppression and POD remains controversial, and studies have contradictory results. This review will explore the current methods used to evaluate burst suppression, its origins and proposed mechanisms, relation to cognitive outcomes, role of medications, and associated risk factors, to present the current understanding of intraoperative burst suppression and its consequences.

## Burst Suppression Detection

Burst suppression is detectable visually in raw EEGs, however, the best methods to study the phenomenon are not established. Standardizing burst suppression measurement is crucial to decreasing variability, as one study found that different interelectrode distances produce different burst durations (Moldovan et al., 2016). Measurement is also challenging since burst suppression patterns are inherently irregular—observed “spectral drifts” in bursts suggest that burst suppression may be more variable rather than a strict on-off switch of the EEG signature (Ching et al., 2012; Lewis et al., 2013; Liley and Walsh, 2013; Shanker et al., 2021).

As a result, commercial technologies estimating burst suppression have emerged and are used as a proxy of burst suppression measurement, leading to prevalent conflation of the two. Some of the commonly used EEG measurements include bispectral index (BIS), WAV_CNS_ (Wavelet Analysis Value for Central Nervous System monitoring), and patient state index (PSI). These indices quantify the patient’s brain activity, providing a 0–100 score (Bistm Complete Monitoring System, 2013; SedLine Sedation Monitor Quick Reference Guide, 2018). In addition, these monitors can calculate burst suppression ratio (BSR) or suppression ratio (SR) presented as the percentage (0–100) of epochs in the last 63 s where the EEG voltage from the frontal and pre-frontal cortex is considered suppressed (Bistm Complete Monitoring System, 2013; Muhlhofer et al., 2017; SedLine Sedation Monitor Quick Reference Guide, 2018). For the BIS, studies had shown that there is a linear relationship with burst suppression ratio (BSR) for BIS values lower than 40 (Bruhn et al., 2000). In addition, studies had shown that while BSR and BIS are linearly correlated for BIS <40%, there is inadequate reflection of anesthetic drug effect for ratios >40%, (Bruhn et al., 2000) and BSR underestimates burst suppression compared to visual analysis of raw data (Muhlhofer et al., 2017). Furthermore, one study observing anesthetic level using a burst suppression estimation tool of state entropy from the GE Entropy Module EEG machine found it is not as connected with the BSR as BIS, so longer episodes may lead to incorrect titrations and anesthetic depth (Georgii et al., 2020). Another study that evaluated different measures of EEG burst suppression to identify the best predictor of POD found that the burst suppression duty cycle (BSDC), a measurement of the proportion of time spent in a burst phase (1-BSR) was a good predictor of POD after cardiac procedures (Ma et al., 2020). Other studies have used scores that incorporate the total brain power spectra density, alpha band power spectral density, and the propofol dose to determine burst suppression occurrences (Touchard et al., 2019). This score is called BP_TIVA_; when low, it is associated with higher suppression time, lower alpha band power, and lower total power, measurements that can provide information about patient’s brain state (Touchard et al., 2019). Another measurement of the brain’s state is the burst suppression probability (BSP), which gives the instantaneous probability of being suppressed at a given time, based on a state-space model algorithm that uses instantaneous burst suppression state (Chemali et al., 2013).

Although machine algorithms correlate with burst suppression and are easier to interpret, they underestimate burst suppression (Muhlhofer et al., 2017). Therefore, measurement with visual analysis of raw EEG data is more accurate to identify periods of burst suppression, though more difficult and requires training (Muhlhofer et al., 2017). Bombardieri and colleagues explored the feasibility of training physicians. They found that a 35-min training resulted in significant improvements in identifying EEG waves for different hypnotic depths and recognizing when the processed EEG index was discordant with the hypnotic depth suggested by the EEG monitor (Bombardieri et al., 2020). As visual analysis of raw EEG waveform is more accurate for burst suppression measurement than algorithms, this could serve as a more precise parameter for the level of anesthesia and may better reduce POD occurrence.

## Mechanism

Studies have yet to determine the mechanism behind burst suppression, though many have used animal models, human data, and mathematical modeling to better understand the underlying neurophysiology.

One important proposition is the cortical hypersensitivity hypothesis. Using a cat model, the authors found that there is a neural response to stimulation during burst suppression (Steriade et al., 1994; Kroeger and Amzica, 2007). They proposed that hyperexcitability comes from increased concentration of extracellular calcium resulting from high doses of isoflurane (Kroeger and Amzica, 2007; Rojas et al., 2008). Burst suppression could then be explained by a post-burst refractory period where the mechanical stimulus depends on the time between the end of a burst and the next stimulus (Steriade et al., 1994; Rojas et al., 2008). There is support for this hypothesis from the consistency in the duration of the refractory period with the time needed for the levels of extracellular calcium to return to baseline after bursting activity (Steriade et al., 1994; Kroeger and Amzica, 2007).

Another prominent theory is the metabolic hypothesis (Ching et al., 2012). Using a mathematical model, the authors showed how burst suppression arises through the interaction between neuronal dynamics and brain metabolism (Ching et al., 2012). In this model, a decrease in cerebral metabolic rate, coupled with stabilization by adenosine triphosphate (ATP)-gated potassium channels, leads to burst suppression (Ching et al., 2012). Under these conditions, there are low levels of ATP, which may lead to protection of membrane integrity and cellular preservation (Forgacs et al., 2020). An important aspect of this hypothesis is that it can be applied to scenarios of decreased metabolic activity such as general anesthesia, hypothermia, and brain injury (Rampil, 1998; Ching et al., 2012; Shanker et al., 2021).

## Intraoperative—Cardiac Surgery

Burst suppression was first studied in cardiac surgeries due to concerns about the impact of cardiopulmonary bypass on the brain. Most studies have found an associative relationship with burst suppression algorithms, such as decreased BIS, leading to postoperative complications. The B-Unaware trial group found the cumulative duration of low BIS to be independently associated with intermediate-term mortality, with a 29% increased risk of death per hour with BIS index less than 45 (Kertai et al., 2010). They also found no relationship with BIS and end-tidal anesthetic gas concentrations during the maintenance phase, volatile anesthetic concentration, or duration of anesthesia (Kertai et al., 2010). However, cognitive outcomes were not explored in this paper or their subsequent 2011 study with a larger cohort of patients, which found no association of mortality with low BIS values (Kertai et al., 2011). In contrast, other studies focused on evaluating postoperative cognitive outcomes in patients undergoing cardiac surgeries. Three different studies demonstrated that patients that had intraoperative EEG burst suppression have increased POD (Soehle et al., 2015; Momeni et al., 2019; Pedemonte et al., 2020). Furthermore, a study on aortic surgeries found that lower BIS values were associated with increased POD and neurological events such as stroke and transient ischemic attack, in addition to increased length of ICU stay and intubation time (Santarpino et al., 2011). Moreover, a study of comatose patients after cardiac arrest in cardiac surgery found that burst suppression with identical bursts was a distinct pathological EEG pattern after diffuse cerebral ischemia that was invariably associated with death (Hofmeijer et al., 2014). Others have suggested that EEG patterns may reflect a dynamic brain state and reveal insights into underlying neurological functions in disorders of consciousness (Sekar et al., 2019; Edlow et al., 2021).

In contrast to the potential detrimental role of burst suppression, some studies suggest burst suppression monitoring has no effect on postoperative cognitive function and burst suppression may be protective. Newman et al. (1995) reported that burst suppression from propofol produced a statistically significant reduction in cerebral blood flow, cerebral oxygen delivery, and cerebral metabolic rate, indicating a neuroprotective potential for burst suppression by reducing cerebral exposure to embolic load. However, a study by Roach and colleagues found no significant difference in neuropsychologic deficits if the patient received or didn’t receive propofol-induced burst suppression (Roach et al., 1999). Furthermore, studies using a rat model of barbiturate-induced burst suppression, hypothermia, and burst suppression plus hypothermia showed reduced infarct volumes of 32, 71, and 66%, respectively, suggesting that burst suppression is not required for additional neuroprotection under hypothermia (Westermaier et al., 2000). It is important to note that burst suppression may arise under hypothermic conditions induced during circulatory arrest to protect the brain from hypoxemic-ischemic damage (Arrica and Bissonnette, 2007; Westover et al., 2015).

## Intraoperative–Non-Cardiac Surgery

Many studies turned to non-cardiac cases to determine whether burst suppression impacts patients in other settings due to the initial data on burst suppression and adverse outcomes from cardiac surgeries. Various human randomized control trials (RCTs) and cohort studies suggest that using EEG indices such as BIS may prevent burst suppression and lead to a decrease in POD. One cohort study in 2003 found that BIS-guided groups and auditory evoked potential (AEP)-guided groups administered less average end-tidal desflurane concentrations. In the BIS-guided group, the extubation times and length of post-anesthesia care unit stay were significantly different from the control group (Recart et al., 2003). An RCT in 2012 of non-cardiac surgeries using a BIS monitored intervention, found that postoperative cognitive decline (POCD) was significantly reduced at 1, 12, and 52 weeks after surgery, with significant improvements in reaction time and executive function (Ballard et al., 2012). Due to the increased BIS values in the BIS-guided group, a 2013 RCT found a lower incidence of POD than the control group, 21.4% compared to 16.7% in the BIS monitored group (Radtke et al., 2013). The relationship of increased time in burst suppression resulting in increased POD, as well as greater end-tidal volatile anesthetic concentration and lower intraoperative opioid dose increasing burst suppression duration, was also reflected in an observational cohort study in 2016 (Fritz et al., 2016). Subsequently, a cohort study in 2018 that used processed hypnograms in conjunction with a visual reading of EEG spectrograms, found that the estimation of EEG burst suppression was predictive of POD particularly in emergence trajectories lacking spindle power–especially with ketamine and nitrous oxide administration (Hesse et al., 2019). These subjects were also at an increased risk for readmission and twice as likely to stay >6 days in the hospital (Hesse et al., 2019). Two 2018 reviews looking at anesthetic titration based on bispectral EEG data found extensive heterogeneity for the studies and significant publication bias (Luo and Zou, 2018; Punjasawadwong et al., 2018). However, they concluded that EEG-guided care significantly reduced the risk of POD and long-term cognitive dysfunction (Luo and Zou, 2018; Punjasawadwong et al., 2018). A study using 1-min EEG epoch analysis on spine surgery patients in 2021, found more prevalent burst suppression in those who experienced POD, but a similar proportion of time in maximal burst suppression between those with and without POD (Lele et al., 2021). Therefore, the study could not conclude a dose-response relationship between burst suppression and POD, although they recommended neuromonitoring and EEG-guided surgery (Lele et al., 2021). Another observational study in 2021 using MT Monitor Technik’s Narcotrend-Compact M (raw EEG) found that occurrence of POD was associated with burst suppression, greater mean arterial pressure (MAP) variance, and sevoflurane concentration (Jung et al., 2021). In contrast, a retrospective study using BIS indices showed that patients that spent less time in burst suppression and deep states were more likely to develop POCD, suggesting that the parameter of burst suppression may be protective for postoperative dysfunction (Deiner et al., 2015).

On the other hand, various studies have not found a decrease in POD using intraoperative EEG indices to minimize burst suppression. A 2002 orthopedic surgery study using BIS monitoring intervention during surgery, demonstrated that groups with average BIS index 44 vs. 51 had no difference in POCD or psychometric tests but found decreased isoflurane titration and faster emergence of elderly patients from anesthesia when the BIS index was used (Wong et al., 2002). As noted, these BIS values were >40 in both groups, which could affect the ability to see any changes that occur at lower BIS values. Furthermore, the 2019 ENGAGES trial evaluated whether EEG-guided anesthetic administration decreased the incidence of postoperative delirium in elderly patients (Wildes et al., 2019). In this case, the investigators instructed the non-blinded group to minimize BIS index values below 40 (Wildes et al., 2019). They evaluated time with BIS <40 and showed that the median cumulative time with EEG suppression was significantly less (7 vs. 13 min) in the EEG-guided group (Wildes et al., 2019). They also found that when the providers were blinded to the EEG monitor, the time spent with BIS values <40 was longer (32 vs. 60 min) (Wildes et al., 2019). However, there were no significant differences in POD among the groups (Wildes et al., 2019). In contrast to the ENGAGES trial, the 2013 CODA study found that BIS-guided anesthesia reduced anesthetic exposure and decreased the risk of POCD (Chan et al., 2013). In this case, they used the BIS values to compare the groups where the BIS group had a higher BIS value that was significantly different from the routine care group (53 vs. 36) (Chan et al., 2013). Similar to the ENGAGES trial, they also calculated the median time spent with BIS <40 which was also statistically lower in the BIS group (7.2 vs. 22.8 min) (Chan et al., 2013). Furthermore, the 2020 ADAPT-2 trial with 204 geriatric patients (mean age 72 ± 5 years) found that using an EEG device to maintain a PSI > 35 decreased time in burst suppression intraoperatively (Tang et al., 2020). Delirium was lower in the interventional group; however, the difference was not statistically significant (Tang et al., 2020).

Due to the varying results from trials, controversy in the field persists regarding the role of burst suppression on POD. Critics of the CODA and ENGAGES trials highlight a significant limitation in the field–underestimation of burst suppression in measurement–which may have occurred with the studies’ cutoff of BIS <40 instead of using raw EEG analysis. The reduction in anesthetic concentrations was also greater in the CODA study as BIS monitoring reduced end-tidal volatile concentrations by 29.7% (0.93–0.57) vs. 0.8–0.69 in the ENGAGES trial, which may have impacted POD occurrence (Chan et al., 2013; Wildes et al., 2019). Overall, the differences among the groups were greater in the CODA trial when evaluated for time with BIS >40 and the BIS values were significantly different among the CODA groups (53 vs. 36). These differences could also account for the differences observed in POD among the studies. Variabilities in anesthetic concentrations, threshold values, and variability in EEG monitoring methods with potentially different sensitivities for burst suppression detection (i.e., estimation by BIS or PSI vs. raw EEG monitoring) may all lead to the discordant results in the field.

## Risk Factors

There are various risk factors for burst suppression, including age, comorbidities, baseline impairment, and sensitivity to medication. Analysis of propofol-remifentanil general anesthesia maintenance in a non-cardiac surgery study found that independent factors associated with SR were advanced age, history of coronary artery disease, and male gender (Besch et al., 2011). Age-related changes in geriatric patients are associated with decreased alpha power (Kratzer et al., 2020), alpha coherence, peak frequency, and increased burst suppression occurrence (Purdon et al., 2015a). Low alpha and beta power have been linked to older age, burst suppression vulnerability, reduced brain metabolism, decreased cognition, and increased risk for postoperative complications such as POD (Plummer et al., 2019; Shao et al., 2020). However, it is important to note that burst suppression could be overestimated due to the changes that result from aging, including low EEG voltage from cortical thinning and reduced brain volume (Purdon et al., 2015a). As geriatric patients were found to have different burst suppression patterns (Kratzer et al., 2020), provider awareness of these differences and the ability to identify these patterns are critical. Because commercial EEG algorithms do not account for age (Purdon et al., 2015a), monitoring unprocessed EEGs may better prevent adverse postoperative effects in geriatric patients. Additionally, even young and middle-aged patients have shown a benefit from BIS-guided anesthesia, as a gynecologic surgical study found that a group maintaining BIS 40–50 had decreased POCD than those with BIS <40 (Shu et al., 2015).

Lastly, anesthetic agents may have different impacts on patients and their EEGs due to differences in mechanisms and patient sensitivities and should be considered in dosing to avoid burst suppression. Medications acting on gamma-aminobutyric acid type A receptors all produce burst suppression at high doses and include halogenated ethers, barbiturates, propofol, etomidate, and N-methyl-D-aspartate receptor antagonists, which all have unique EEG signatures (Akrawi et al., 1996; Huotari et al., 2004; Brown et al., 2011; Akeju et al., 2016; Hambrecht-Wiedbusch et al., 2017; Fleischmann et al., 2018). Halothane has minor effects on the EEG and does not cause burst suppression even at high doses (Antunes et al., 2003; Murrell et al., 2008). However, patients that have increased sensitivity to volatile anesthetics, and a history of smoking, present with EEG suppression at lower anesthetic concentrations and have higher incidence of POD (Fritz et al., 2018). In addition, others have reported risk factors for EEG suppression that lead to increased postoperative mortality; these included older age, number of comorbidities, and higher intraoperative volatile anesthetic, opioid, or benzodiazepine use (Willingham et al., 2014).

## Discussion and Future Directions

As we continue to understand the role of intraoperative burst suppression in the development of POD, it is crucial to learn from the factors that may have led to incongruencies in prior studies. Despite significant progress in the field, there is no gold standard that establishes the best system to monitor burst suppression, underlying mechanisms of burst suppression, the role of age, or patient’s risk factors in the development of burst suppression. While studies show processed EEG values underestimate burst suppression, most studies conflate burst suppression with BIS or PSI values. Moreover, induction and maintenance burst suppression occurrence is not typically separated in analysis, which may be a confounding factor. Raw EEG-guided intraoperative drug titration may be a better alternative to detect and prevent burst suppression and downstream POD with proper training for providers. Furthermore, the factors that constitute a vulnerable brain are yet to be established, which will help better evaluate risk for burst suppression. Aside from age and baseline cognitive function, factors such as traumatic brain injuries, comorbidities, mental health diagnoses, and socioeconomic factors are yet to be understood in relation to burst suppression. Monitoring those with decreased baseline cognitive reserve may be the best strategy to maximize resources and improve postoperative outcomes. Limitations in addressing these questions include the ethical inability to induce burst suppression in patients and difficulties translating animal studies to the clinical setting. As a result, studies cannot establish a causative relationship between burst suppression and negative outcomes, but rather an association.

## Author Contributions

NP and OBC conceptualized and wrote the manuscript. Both authors contributed to the article and approved the submitted version.

## Conflict of Interest

OBC is an investigator for the clinical trial OLIVER from Medtronic. The remaining author declares that the research was conducted in the absence of any commercial or financial relationships that could be construed as a potential conflict of interest.

## Publisher’s Note

All claims expressed in this article are solely those of the authors and do not necessarily represent those of their affiliated organizations, or those of the publisher, the editors and the reviewers. Any product that may be evaluated in this article, or claim that may be made by its manufacturer, is not guaranteed or endorsed by the publisher.
